# Bishops and the Birth-Rate

**Published:** 1920-09-04

**Authors:** 


### THE PUBLIC WELL-BEING.

### Bishops and the Birth-Rate.

Some very trenchant criticisms have been levelled at that section of the report of the famous Lambeth Conference of Bishops which relates to the problems of marriage and sexual morality; and the attention both of the public and of experts in theology, economics, and eugenics has been more particularly concentrated on the question of the limitation of the birth-rate. Dean Inge published in the *Evening Standard* a remarkably outspoken condemnation of the report. The bishops, it will be remembered, speak of birth control as a practice hostile to the family, and declare that the lowered birth-rate "sounds a note of serious alarm and warning." They go so far as to express the opinion that no means of limiting the family is justifiable, except a complete cessation of marital relations between husband and wife. In this case, Dean Inge roundly asserts, it is impossible to acquit the married bishops of want of candour and courage in recognising facts which are matters of common knowledge.

The causes, as he points out, which have made it necessary for nearly all married people to restrict the number of their children in order that they may do their best for those children who are born, are almost too well known to need recapitulation. It is probably only among the reckless and degraded population of the slums that restriction is not practised, and "only a few very foolish persons think it is either immoral or regrettable." He adds that this voluntary limitation of birth is most marked in the learned professions, who find it increasingly difficult to keep up a decent standard of living; and he states what is undoubtedly the fact, that the clergy themselves are now among the foremost in exercising the necessary prudence. The resulting fall of about thirty per cent. in the birth-rate, which has accompanied the almost exactly parallel fall in the death-rate, still leaves, he thinks, an ample margin of increase.

Mr. Harold Cox, whose views are familiar, attacks the report from another point of view in a published article in which he pictures the ultimate horrors of a totally unrestricted birth-rate. "The rate of increase in our population down to 1911 was so great that were the rate to be maintained for another 360 years the population of England and Wales alone would be immensely greater than the present population of the whole world." He accordingly argues that it is obvious that there must be some limitation not only to our potential rate of increase, but to the actual rate of increase prevailing before the war. "Yet since the war there has been an enormous expansion of the birth-rate, while the death-rate still remains at a low figure. The result is that the increase of the population of England and Wales by excess of births over deaths in the first six months of the present year was greater than any previous period in our history. Such a fact sufficiently disposes of the delusion that our race is in danger of extinction."

It is not necessary to go all the way with Mr. Cox in his argument or to accept final significance of his statistical speculations (which leave out many important human factors) in order to realise that, whatever the bishops may say or Commissioners report, the economic circumstances of the present day are clearly compelling large elements in the community to exercise stringent care in the matter of birth control. It is too obvious to enlarge upon, and denunciations will have no effect when the urgency is so real and pressing. Perhaps the most unfortunate feature of this now common phenomenon is that the persons who are deliberately restricting the size of their families are drawn from those classes which usually produce the strongest children and those most likely to develop into healthy and intelligent citizens. The diminution is least to be remarked in slum areas, partly for the reason that the slum-dweller lives without foresight or care for the future, or a sense of responsibility to casually begotten children; but partly also because the steadily increasing knowledge as to modern methods of birth control has not yet penetrated to their stratum of society. When the knowledge is universal the practice will in time no doubt become universal.

